# Modifying the thickness, pore size, and composition of diatom frustule in *Craspedostauros sp.* with Al^3+^ ions

**DOI:** 10.1038/s41598-020-76318-5

**Published:** 2020-11-11

**Authors:** Mohammad Soleimani, Luco Rutten, Sai Prakash Maddala, Hanglong Wu, E. Deniz Eren, Brahim Mezari, Ingeborg Schreur-Piet, Heiner Friedrich, Rolf A. T. M. van Benthem

**Affiliations:** 1 Laboratory of Physical Chemistry, and Center for Multiscale Electron Microscopy, Department of Chemical Engineering and Chemistry, Eindhoven University of Technology, Groene Loper 5, 5612 AE Eindhoven, The Netherlands; 2 Laboratory for Inorganic Materials and Catalysis, Department of Chemical Engineering and Chemistry, Eindhoven University of Technology, P.O. Box 513, 5600 MB Eindhoven, The Netherlands; 3 Institute for Complex Molecular Systems, Eindhoven University of Technology, Groene Loper 5, 5612 AE Eindhoven, The Netherlands

**Keywords:** Materials science, Biomineralization

## Abstract

Diatoms are unicellular photosynthetic algae that produce a silica exoskeleton (frustule) which exposes a highly ordered nano to micro scale morphology. In recent years there has been a growing interest in modifying diatom frustules for technological applications. This is achieved by adding non-essential metals to the growth medium of diatoms which in turn modifies morphology, composition, and resulting properties of the frustule. Here, we investigate the frustule formation in diatom *Craspedostauros sp.*, including changes to overall morphology, silica thickness, and composition, in the presence of Al^3+^ ions at different concentrations. Our results show that in the presence of Al^3+^ the total silica uptake from the growth medium increases, although a decrease in the growth rate is observed. This leads to a higher inorganic content per diatom resulting in a decreased pore diameter and a thicker frustule as evidenced by electron microscopy. Furthermore, ^27^Al solid-state NMR, FIB-SEM, and EDS results confirm that Al^3+^ becomes incorporated into the frustule during the silicification process, thus, improving hydrolysis resistance. This approach may be extended to a broad range of elements and diatom species towards the scalable production of silica materials with tunable hierarchical morphology and chemical composition.

## Introduction

Unicellular photosynthetic algae commonly known as diatoms are ubiquitous throughout most aquatic environments^1^. As such diatoms have evolved to tolerate or adapt to a broad range of environmental conditions including the presence of non-essential metals ions, some of which are toxic^2,3^. The most characteristic feature of diatoms are their biogenic silica cell walls termed the frustule comprising two valves (epitheca and hypotheca) and a number of girdle bands^4^. The frustule, which has a species-specific morphology including micro and nanopatterns, is reproduced during the cell division cycle^5^. For frustule formation in each cycle, water-soluble monosilicic acid, a silica precursor, is taken up from the environment into the diatom cell by silicic acid transporter proteins (SITs)^6,7^. Eventually, the frustule is constructed in a confined intracellular organelle with mildly acidic pH called the silica deposition vesicle (SDV)^8^. The presence of some specific macromolecules such as silaffines and long-chain polyamines in the frustule has led to the assumption that they are involved in silica formation^9,10^. However, the intracellular processes which ultimately lead to frustule formation are remarkably altered in response to changing environmental conditions^11^.

Besides salinity, light intensity/wavelength, pH and temperature^12–17^, also the presence of non-essential metals^18^ is influencing the chemistry and morphology of frustule formation. Diatoms can take up some of these elements into the silica, thus, modifying the frustule properties^19^. For instance, the presence of germanium in the growth medium of *Craspedostauros sp.* has led to an alteration in the pore array morphology and induced photoluminescence and electroluminescence properties^20^. Similarly, cadmium uptake by *Nitzschia palea* not only has modified the morphology of the costa and pores of the valves but also altered the embedded organic constituent^21^. Furthermore, the addition of titanium, zirconium, tin, and germanium to the growth medium resulted in variations in frustule morphology of *Synedra Acus* adversely affecting on the mechanical strength of the valves^22^. Lastly, incorporation of titanium by *Thalassiosira weissflogii* has resulted in photocatalytic activity of the frustule material^23^.

Another technologically important element is aluminum (Al) that can be taken up and incorporated into the frustule of diatom modifying its properties^24^. Incorporation of Al into the *Thalassiosira pseudonana* with a silicon to aluminum (Si:Al) ratio of 50:1 has resulted in catalytic activity^25^. Most notably, Al incorporation into frustules of several diatom species has shown a significantly enhanced hydrolysis resistance compared to their Al-free types^26,27^. To date, the internalization of Al into the diatom cells has been investigated from a biological perspective in which Al is distributed within the cell at different subcellular compounds such as granules, debris, organelles, and proteins^28,29^. From the material science perspective exploring the effect of inorganic constituents like Al on the frustule pore size or thickness and the parameters controlling silicification are important too. In recent years, there has been an increasing interest in employing diatom frustules for technological applications such as catalysts, in optic, for drug delivery, and for separation applications^30–35^. The morphological features of the nanopatterned structures are crucial for some of these applications. However, these technologically interesting effects of Al on frustule formation including morphology, thickness and composition have never been studied.

In this manuscript, the effects of Al^3+^ on the biogenic silica formation in raphid pennate marine diatom *Craspedostauros sp.* (*C. sp.*) were studied. Besides cell density and total silicic acid uptake in dependence of Al^3+^ concentration in the growth medium, especially morphological changes, thickness variations and composition were studied by a combination of imaging and spectroscopy techniques. This investigation has unveiled that not only *C. sp.* responded to the presence of Al^3+^ via decreasing the growth rate and increasing Si uptake, but also there was an interdependence between the Al^3+^ concentration and nanoporosity as well as the thickness of the valve. Unraveling the correlation between Al^3+^ ion concentration and morphological features is a crucial step towards manipulating the nanopatterned structures in diatoms for technological applications.

## Materials and methods

### Diatom culture

*C. sp.* (UTEX B679) was received from the UTEX Culture Collection of Algae and cultivated in artificial seawater medium supplemented with f/2 medium (ASW-f/2). The chemical composition of the medium is provided in the Supplementary Table S1. The cell cultures were synchronized using the following procedure, cells of *C. sp.* were grown inside a climate cabinet (Flohr, Netherlands) at 23 °C, a cycle of 14 h day/10 h night, and light intensity of 3000 lx. Small aliquot (1 ml) (depending on the required volume of the final culture) of a stock culture was added to a flask culture which contained ASW-f/2 medium. After seven days cells were gently harvested via centrifugation at 2.4 rpm and incubated in silicon free medium (contained aforementioned ASW-f/2 components except for silicon) for at least 24 h to have a synchronized starter culture.

### Al^3+^ addition experiments

Different aluminum concentrations were employed in the form of dissolved aluminum chloride in artificial seawater to achieve Al^3+^ concentrations of 0.2 µM, 1 µM, and 2 µM which resemble the upper level of concentrations found in the seawater. This naturally occurring upper concentration of Al^3+^ is found in the tropical Angola Basin and the inlet of the Amazon river and is the concentration found in coastal regions^27,36,37^. A control culture was grown without Al^3+^ addition. Four batches of *C. sp.* cultures were prepared as follows, 20 mL of ASW-f/2 and desired Al^3+^ concentration in a 75 mL culture flask was inoculated with 0.5 mL of the synchronized starter culture. Culture flasks were shaken manually every single day to assure proper mixing of the medium. Throughout this paper, the terms ‘C_0_, C_1_, C_2_, and C_3_′ will refer to diatom cultures with 0, 0.2, 1, and 2 µM Al^3+^ ion concentration, respectively.

### Cell density measurement, Si and Al^3+^ uptake, and silica content

Cell density measurements were performed using an Automated Cell Counter for three biological replicates. The growth rate within the exponential phase was calculated using the following equation $\mu = \left[ {ln\left( {N_{f} } \right) - ln\left( {N_{i} } \right) } \right]/t$, where N_f_ and N_i_ are final and initial cell numbers respectively, and t is test period in day^38^. Silicon (Si) and Aluminium (Al) uptake was measured using an atomic absorption spectrometer (Shimadzu AA7000, Japan) equipped with a GFA 7000 pyrolytic coated furnace tube, an autosampler, and a D2 background correction lamp. The wavelengths employed for Al and Si measurements were 309.5 nm and 251.8 nm, respectively. To measure the concentrations at the different interval times in the growth medium, diatom cells were filtered out by centrifugation. At each time point, 500 µL of the medium was extracted and filtered with an Amicon Ultra-0.5 centrifuge filter (5 min, 14,500 rpm). 100 µL of the filtrate was diluted 100 times with Milli-Q and subsequently used for the measurements. To prevent silica contamination no glass ware was used throughout the whole process. By combining the results of cell density and Si uptake we could estimate the silica content per diatom cell. Therefore, the silica content per cell was calculated via dividing the amount of consumed Si in the growth medium by the number of cells that have been grown until day 4 which corresponds to the maximum cell density in the four cultures. Statistical significance was measured using a two-tailed *t* test.

### Sample preparation for electron microscopy

Samples for electron microscopy were prepared in the following manner. First, brown diatom pellets were isolated from the suspended cultures at 14,500 rpm via centrifugation (Minispin Centrifuge, Eppendorf, Germany). The centrifuges pellets were suspended in Milli Q water and centrifuged. The process was repeated 5 times in order to ensure complete removal of the salts, unreacted silicic acid and obtain a brown pellet. Common procedure for removing organic constituents of diatom frustule involves extraction using EDTA–SDS may impact the composition of the extracted frustule due to the enhancement of the solubility of silica in the presence of EDTA^39^. Intracellular components were extracted using ethanol in order to minimize the impact of extraction on chemistry and morphology of the frustule. The brown pellets were dispersed in absolute ethanol and centrifuged at 14,500 rpm to split girdle bands and valves form each other and to remove the organic constituents. This procedure was repeated at least 10 times to obtain an off white pellet at the bottom of the centrifuge tube. In order to ensure the complete isolation of valves from girdle bands, a suspension of the pellet was sonicated in a bath sonicator (Bransonic ultrasonic cleaner, model 1510E-DTH, 42 kHz, USA) for 5 min.

### Scanning electron microscopy (SEM) imaging and energy-dispersive X-ray spectroscopy (EDS) measurements

For SEM imaging, a tiny amount of the white pellet was dispersed in ethanol and 50 µl of the suspension was dropped onto a silicon wafer that was fixed to a standard SEM stub followed by drying in air. The material was then sputter-coated with gold using Turbo sputter coater Emitech K585X Dual (UK) with a thickness about 10 nm. For EDS line scan and elemental mapping, 10 µl of the above suspension was pipetted onto a standard 200 mesh copper TEM grid covered by a 10 nm continuous carbon film. TEM grids were placed in an in-house made sample holder suitable for a SEM stage. SEM–EDS line scan and mapping were performed using SEM Quanta 3D FEG (Thermo Fisher Scientific, USA), at an acceleration voltage of 3 and 10 kV. EDS spot measurements were performed at an accelerating voltage of 10 kV using Phenom proX SEM (Thermo Fisher Scientific, USA). The mean diameters of areolae, and small pores were calculated via in house MATLAB scripts as shown in the Supplementary Figs S1, S2. In addition, length and width of the valves and morphological features such as transapical rib, cross extension, distance between neighboring areolae within an array (Da) and distance between two areolae of two parallel arrays (Dp) were measured using Gatan Digital Micrograph for at least 15 valves pre culture. The area of the valve was determined as an ellipse.

### Thickness measurements and the volume of the frustule

TEM images were acquired on a Tecnai T20 G2 (Thermo Fisher Scientific, USA) operated at 200 kV and equipped with a LaB_6_ filament and a 4 k CETA CCD camera. All the diatoms were imaged at the same magnification of 800 × with a typical dose < 0.5 e^−^.nm^−2^ s. Thickness mapping of diatoms was carried out with an in house MATLAB script which is detailed in the supplementary information (Supplementary Table S2 and Supplementary Fig. S3). All scripts were developed in MATLAB version 2018b, https://nl.mathworks.com/products/matlab.html. Furthermore, the details of the calculated frustule volume are presented in the supplementary information (Supplementary Fig. S4). Statistical significance was measured using a two-tailed *t* test.

### FIB-SEM and internal view of *C. sp*

In order to get some insights into how *C. sp.* responds to the presence of Al^3+^ ions, internal structures of the cell were exposed via focused ion beam scanning electron microscopy (FIB-SEM). Thus, *C. sp.* cells were harvested by centrifugation and fixed with 2.0% paraformaldehyde and 1% glutaraldehyde for 2 h at room temperature. The fixed cells were gently rinsed two times with 0.1 M sodium cacodylate buffer (pH 7.2) and postfixed for 1 h at room temperature with 0.5% (w/v) OsO_4_. Then stained in 0.1% uranyl acetate for 3 h. After the staining cells were rinsed four times with Milli-Q water. The dehydration of the fixed cells was performed in an upgraded sequence of ethanol. Ultimately, Epon 812 was used to embed the cells. FIB tomography was typically conducted utilizing the aforementioned SEM. Throughout the FIB process, a beam of focused ions (Ga^+^) removes 100 nm thick slice from the surface of the embedded *C. sp.* cell at a current of 1 nA and acceleration voltage of 30 kV. Subsequently, an image of the freshly revealed surface was taken at an accelerating voltage of 5 kV by Back-Scattered Electron detector (BSE). This process was repeated slice by slice throughout the entire embedded cell (Supplementary Fig. S5 shows an overview of the process). Avizo (9.5, Thermo Fisher Scientific, USA) software was used for alignment, segmentation and rendering of the internal features of *C. sp.*

### NMR spectroscopy and TGA

^27^Al solid state NMR spectra on diatoms was recorded using a Bruker DMX500 Avance spectrometer (Germany) operating at ^27^Al resonance frequency of 130.3 MHz. The measurement was performed at 25 kHz rotor spinning rate using a triple resonance 2.5 mm MAS NMR probe head. An one-pulse sequence was used with an 18° pulse of 1 µs duration and an interscan delay of 0.5 s. ^27^Al chemical shift was calibrated using saturated Aluminium nitrate solution. TGA measurements were performed using Q500 TGA (TA instruments, Germany). Diatom pellets (2 to 4 mg) were loaded on to platinum pans and the heated from 30 °C to 1000 °C in air. The temperature was ramped up at 20 °C/min while maintaining a gas flow rate of 60 mL/min.

### Frustule dissolution experiment

In order to monitor the effect of Al^3+^ on the solubility of *C. sp.* frustules, dissolution experiments on C_0_ and C_3_ were carried out. After harvesting and washing the cells with demineralized water, the pellets were transferred into 40 ml demineralized water at 90 °C for 6 days. For measuring the Si concentration samples were taken daily. The sample preparation for Si concentration measurement via atomic absorption spectroscopy was the same as above.

## Results

### Morphological parameters

**Figure 1:**
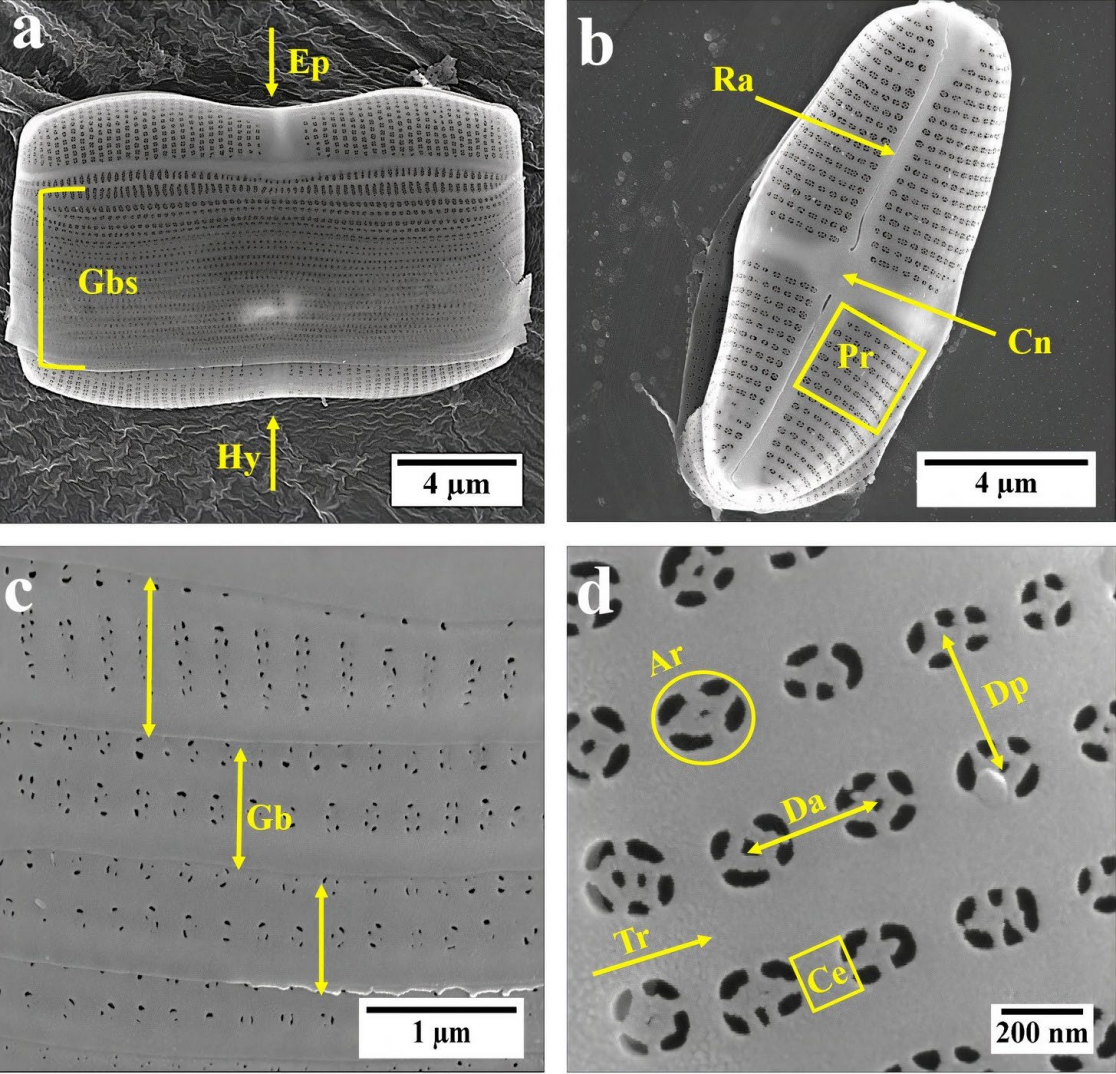
(**a**) SEM images of isolated mature frustule of *C. sp.*; Gbs = girdle bands; Ep = Epitheca (bigger valve); Hy = Hypotheca (smaller valve); (**b**) isolated valve; Ra = raphe; Cn = central nodule; Pr = porous area; (**c**) individual girdle band (Gb); (**d**) porous area, Tr = transapical rib; Ar = areola Ce = cross extension; Da = distance between neighboring areolae within an array; Dp = distance between two areolae of two parallel arrays.

*C. sp.* is a raphid pennate marine diatom that has an imperfect rectangle prism shape. As shown in Fig. 1a, two valves (the epitheca and hypotheca) are connected by a series of overlapping girdle bands. Each valve comprises a thick siliceous structure in the middle known as central nodule combining with raphe rib which elongates throughout the valve resulting in a cross-shaped structure (Fig. 1b). In contrast to the valve, girdle bands are less elaborated and compose of different porous and nonporous areas (Fig. 1c). Within the porous area of the valve, parallel adjacent transapical ribs are connected by neighboring cross extensions to form several arrays of the areolae which are about 200 nm in diameter. The cross extensions bridging two areolae within an array and transapical ribs separating two rows of areolae. Small pores are located within the areolae which have diameter in the range of 60–80 nm (Fig. 1d).

### Morphological changes of *C. sp.* with increasing Al^3+^ concentration

**Figure 2:**
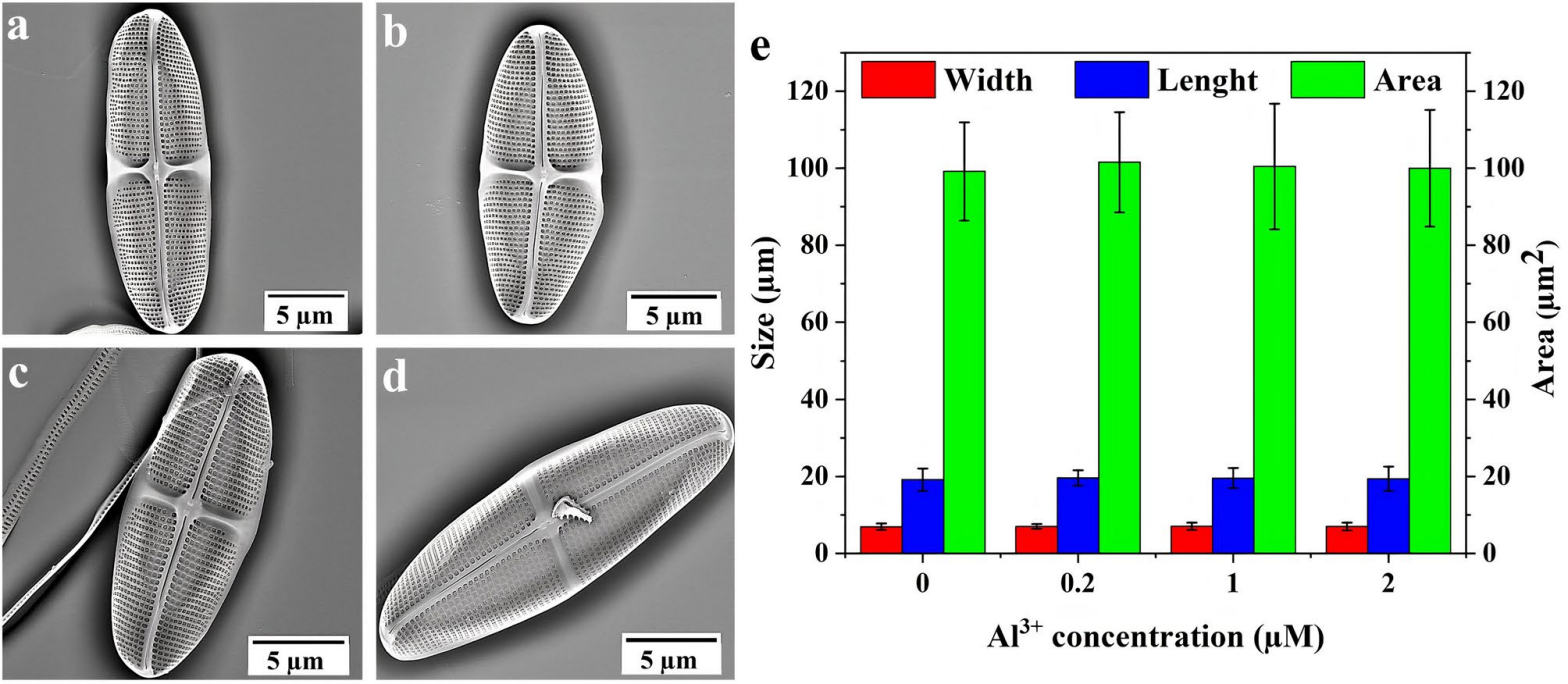
SEM images of the interior surface of the valves of *C. sp.*; (**a**) C_0_; (**b**) C_1_; (**c**) C_2_; (**d**) C_3_; (**e**) measured length, width and area of valves as a function of Al^3+^ concentration obtained from n = 15 valves per culture. Error bars indicate standard deviations.

First it was determined whether morphological characteristics of the valve were modified by the presence of Al^3+^. Figure 2a–d exhibit SEM images of valves grown at different Al^3+^ concentrations. By measuring the length, width, and area of valves it could be shown (Fig. 2e) that the overall size of valves were unaltered due to the presence of Al^3+^.

**Figure 3:**
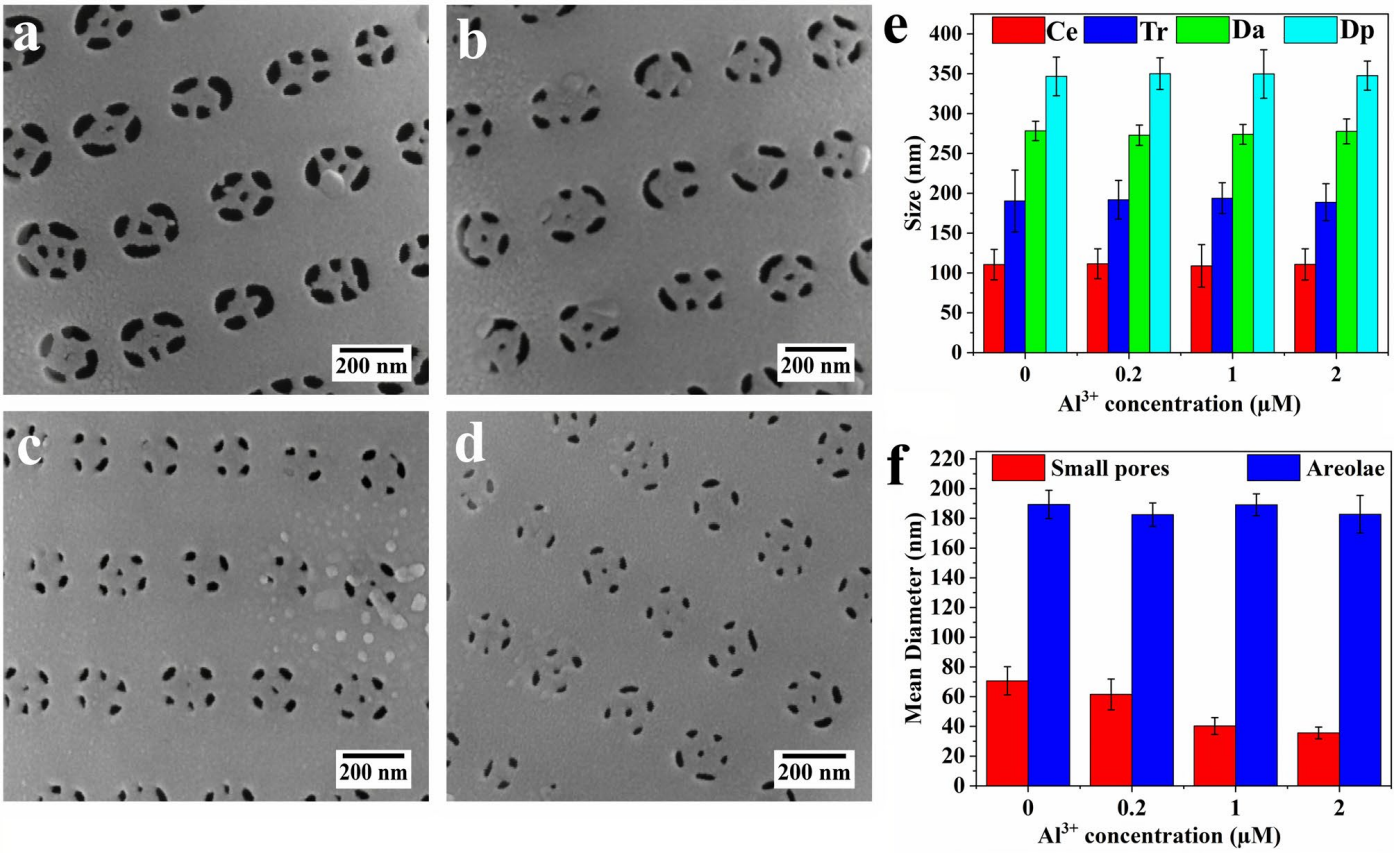
(**a**–**d**) SEM images of porous area of valves of *C. sp.* grown in the cultures with different Al^3+^ concentrations (**a**) C_0_; (**b**) C_1_; (**c**) C_2_; (**d**) C_3_; (**e**, **f**) Bar graphs of measured architectural parameters of *C. sp.* (n = 15 valves per culture) as given in Fig. 1; (**e**) Width measurement of transapical rib and cross extension, distance between neighboring areolae within a pore array and distance between two areolae of two parallel arrays; (**f**) mean diameter of small pores and areolae. Error bars indicate standard deviations.

Nevertheless, a more detailed analysis elucidating the effect of Al^3+^ on the valve architecture at the nano and micro scale indicated subtle differences. Figure 3a–f present high magnification SEM images and a detailed analysis of the porous area. While the overall geometry of the pore arrays remained unchanged with increasing Al^3+^ concentration, i.e., the width of the cross extension (Ce), the width of transapical ribs (Tr), the average distance of the neighboring areolae within a pore array (Da) and the distance between two areolae of two parallel arrays (Dp) stayed constant; what stands out is that the diameter of small pores differs greatly between cultures. There was a close correlation between the reduction of the diameter of small pores and the increase in Al^3+^ ion concentration in the growth medium. The mean diameters of the small pores of the valve of C_0_, C_1_, C_2_, and C_3_ were 70 ± 9, 61 ± 10, 40 ± 5, and 35 ± 4 nm, respectively. Supplementary Table S4 shows the statistical data of the image analysis.

### Macroscopic Al distribution and atomic coordination environment

**Figure 4:**
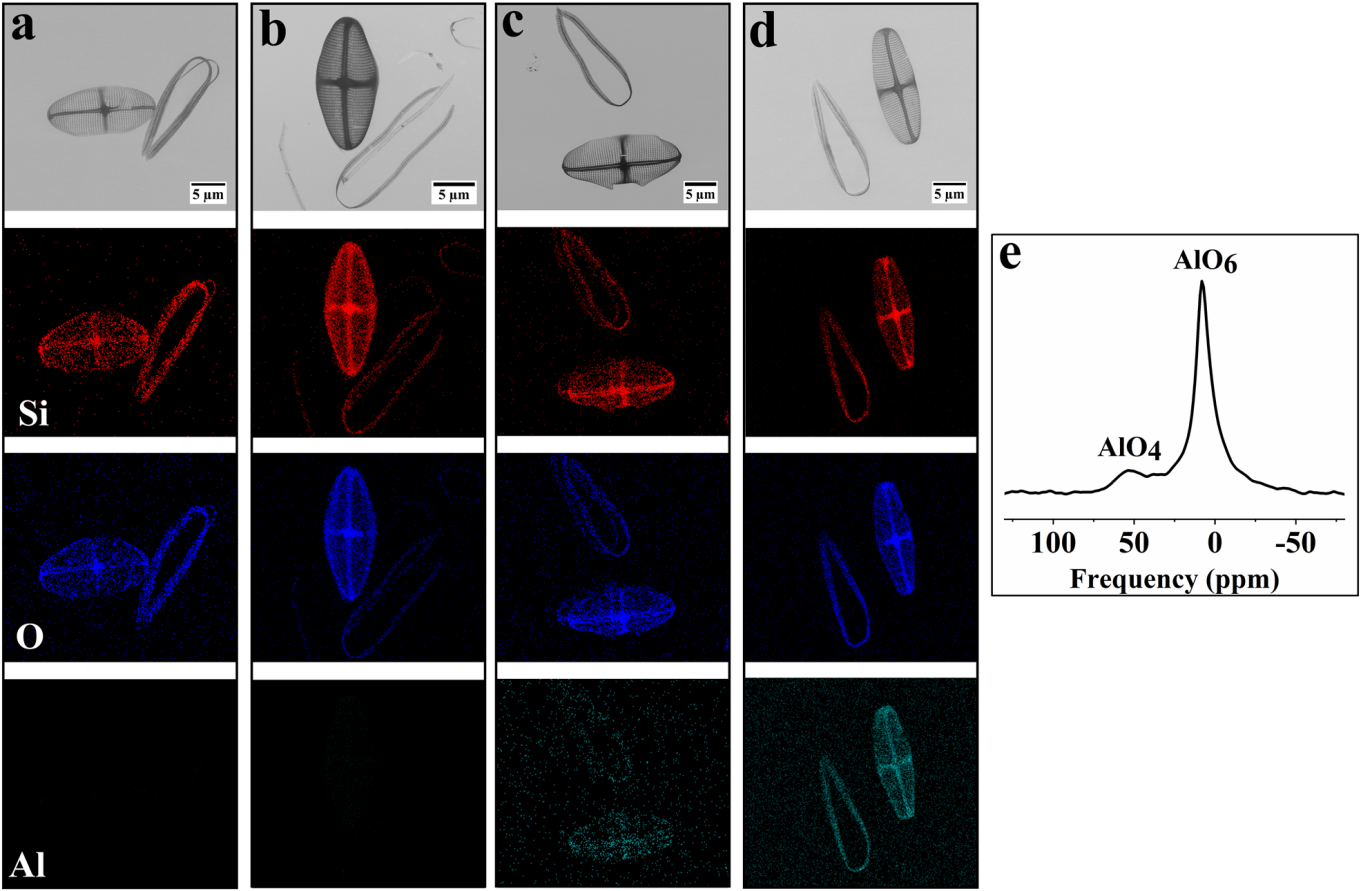
SEM images and EDS elemental maps of isolated valves and girdle bands of (**a**) C_0_; (**b**) C_1_; (**c**) C_2_; (**d**) C_3_; red = Si; blue = O; green = Al; (**e**) ^27^Al solid state NMR of C_3_.

Besides subtle morphological changes, incorporation of Al^3+^ into the frustule may occur. In order to identify the presence of Al in the frustule and how it is coordinated, EDS spot scan, line scan and elemental mapping and ^27^Al solid state NMR of the frustule of *C. sp.* of C_3_ were performed. This sample was chosen for its high concentration of Al^3+^ which can be detected via SEM–EDS and NMR. Also, SEM–EDS spot measurements and mapping of C_0_, C_1_, and C_2_ were conducted to pinpoint the presence and distribution of Si, O, and Al in the valves and girdle bands. Due to the lack or very low concentration of Al in C_0_ and C_1_ EDS did not reveal the presence of Al (Fig. 4a,b and Supplementary Fig. S6). In contrast, mapping of the isolated valves and girdle bands of C_2_ and C_3_ confirmed the presence of Al alongside Si and O (Fig. 4c,d). As shown in Fig. 4d there was a homogenous distribution of Al in valve and girdle bands of C_3_. However, C_2_ did not show a homogenous distribution of Al, which could be due to the low concentration of Al in the sample (~ 100Si:1Al). EDS spot analysis of C_3_ revealed the presence of Si, O, Al, C, Mg, Na, and N. In order to semi quantitatively compare the Si:Al ratios at the submicron level of C_3_, EDS line scans of the porous area and girdle bands were conducted for 50 spots (Supplementary Fig. S6). The mean ratio of Si:Al was approximately 45:1 for both selected features which is comparable to the fed Si:Al ratio of this sample. Solid state ^27^Al NMR measurement was performed to obtain information about the coordination state of aluminum in the frustule (Fig. 4e). The sample showed two well resolved but broadened peaks located at 50 ppm and 6.5 ppm corresponding to four and six coordinated aluminum species^24^. The broad peaks indicate that the chemical environment of aluminum exhibits great structural variability. The presence of Al in the silica matrix has been shown to increase the acidity of synthetic amorphous silica^40^.

### The effect of Al^3+^ on silicon uptake, cell density and total inorganic content

**Figure 5:**
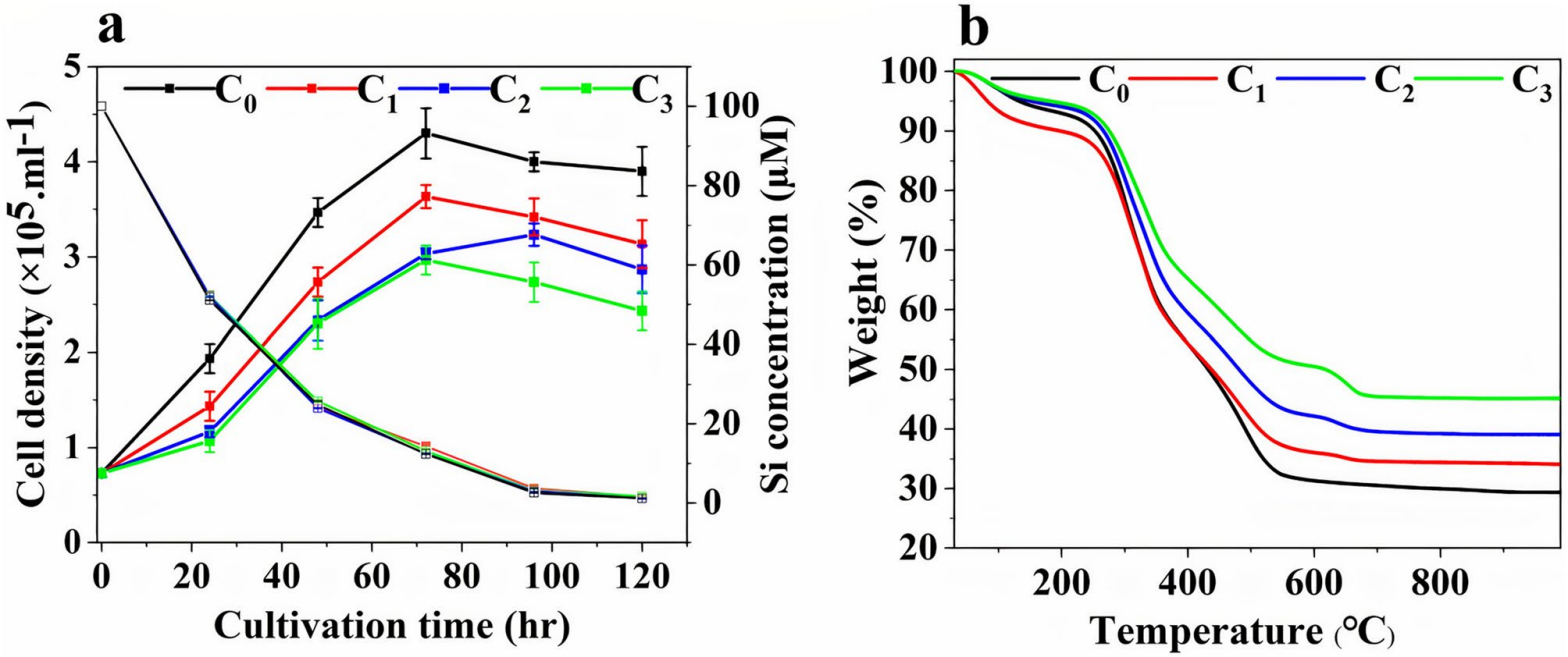
(**a**) Cell density measurements of *C. sp.* at different Al^3+^ concentrations as a function of cultivation time and Atomic absorption spectroscopy of decreasing silicon concentration in the different culture media as a function of cultivation time. Error bars indicate standard deviations for three culture replicates. On day 4th *p* ≤ 0.05. (**b**) Thermogravimetric analysis (TGA) in air, of *C. sp.* frustule material grown at different Al^3+^ concentrations.

To assess whether and how Al^3+^ influences the biosilicification process which ultimately leads to a homogeneous distribution of Al and only altering the pore size*,* Si and Al^3+^ uptake, growth rate and inorganic/silica content of cell cultures were determined. The Si uptake versus cell density measurements of the cultures of *C. sp.* with different Al^3+^ concentrations are shown in Fig. 5a. The figure shows that the cell density gradually increased in all four cultures until day 4 where they reached the maximum cell density. What is striking in this figure, is that the growth rate of the cells decreased by increasing the Al^3+^ concentration in the growth medium also shown in Table 1. Interestingly, Si uptake determined from atomic adsorption spectroscopy showed that the amount of silicon taken up remained unchanged (Fig. 5a). For instance, on day 5, the amount of consumed Si from C_0_ to C_3_ were 96.3 ± 0.2, 96.9 ± 0.1, 97 ± 0.5, and 96.9 ± 0.1 µM, respectively. This implies that increasing the Al^3+^ concentration resulted in diatoms taking up larger quantities of Si per cell. The silica content per cell was estimated by dividing the amount of consumed Si in the growth medium by the number of grown cells, as shown in Table 1. The average silica content of diatom cells for the cultures from C_0_ to C_3_ were 244 ± 7, 298 ± 11, 390 ± 15 and 395 ± 13 fmol/cell, respectively. This equals an increase of about 60% in silica content in the presence of 2 µM Al^3+^ ions. Comparing Al^3+^ and Si uptake for C_3_ showed the same progression for the first 48 h (Supplementary Fig. S7). However, gradually Al^3+^ uptake slowed down compared to Si uptake during the cultivation period which could be due to differences in uptake mechanism^41^. To rule out the possibility of Si precipitation/adsorbtion onto the surface of the cultures flask, a control experiment was carried out for C_3_ without *C. sp.* cells (Supplementary Fig. S8). In addition, in order to verify whether the increased uptake resulted in an increase in the inorganic content, TGA measurements were performed on untreated diatoms by heating them in air from 30 to 1000 °C (Fig. 5b)^42^. The initial 3 to 7% weight loss observed below 200 °C was assigned to adsorbed water molecules^43^. All samples show a steep weight loss between 200 to 550 °C, corresponding to the loss due to the presence of biomolecules molecules such as lipids, proteins, and silanols^44,45^. The samples containing aluminium show an additional weight loss step between 550 and 700 °C. No further weight loss was observed above 700 °C. As shown in Fig. 5b the final inorganic content of the diatoms grown in the absence of aluminium (C_0_) was 29%, whereas the presence of aluminium resulted in an increased inorganic content, namely 47% in total for C_3_. This means that there is an approximately 60% relative increase in inorganic content from C_0_ to C_3_, in close agreement with the results from atomic absorption spectroscopy.

**Table 1:** Growth rate and calculated silica content of *C. sp.* grown in the presence of various Al^3+^ concentration.

Culture number	Growth rate (d^−1^)	Silica content (fmol cell^−1^)
C_0_	0.6 ± 0.05	244 ± 7
C_1_	0.5 ± 0.06	298 ± 11
C_2_	0.4 ± 0.04	390 ± 15
C_3_	0.37 ± 0.03	395 ± 13

Average ± standard deviation calculated from three culture replicates is given.

### The effect of Al^3+^ on valve thickness

**Figure 6:**
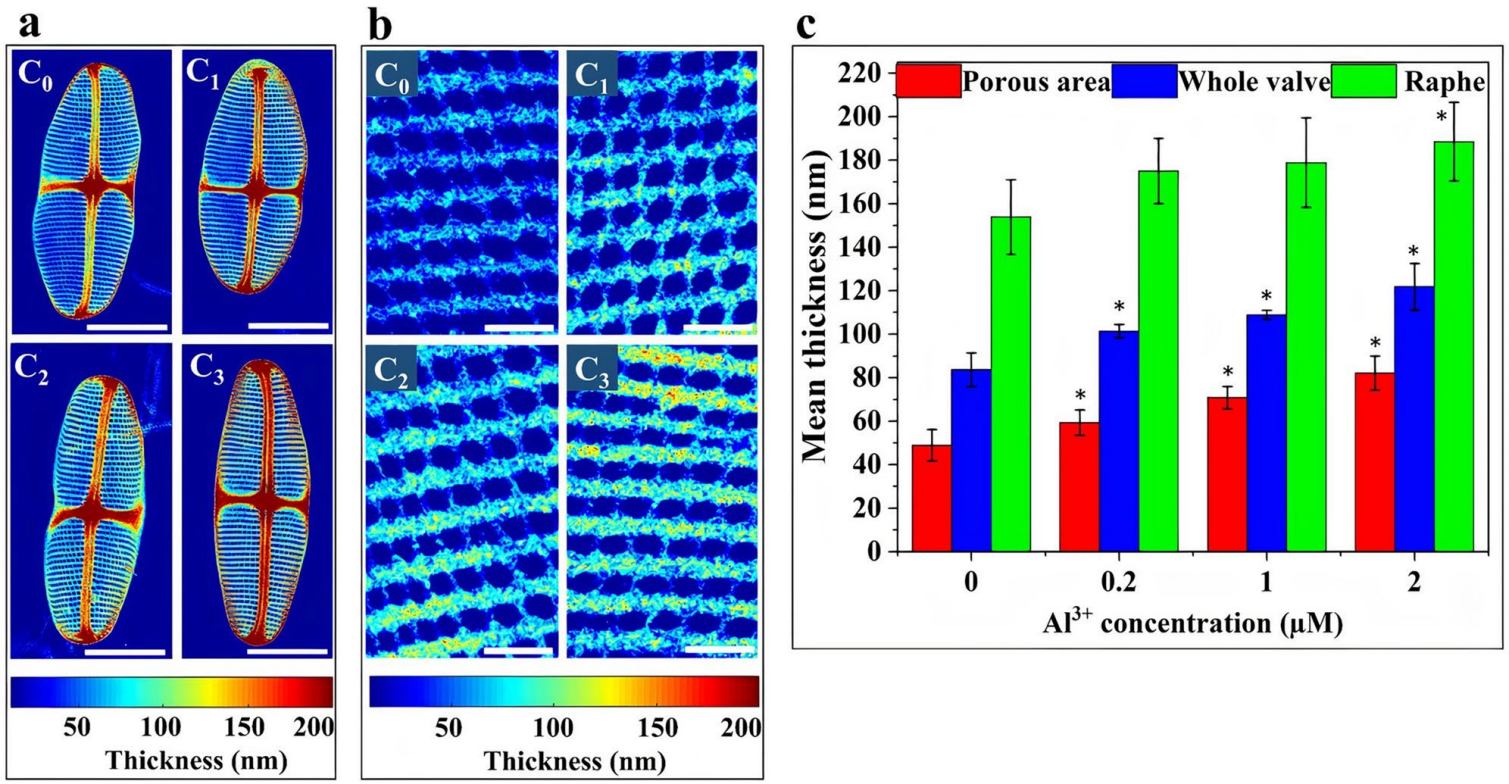
(**a**) TEM thickness maps of *C. sp.* valves grown at different Al^3+^ concentrations (scale bar = 5 µm); (**b**) thickness maps of porous areas (scale bar = 500 nm); (**c**) bar graphs of thickness measurements of porous area, raphe and whole valve (n = 5 valves per culture). Error bars indicate standard deviations.* Indicates *p* ≤ 0.05.

As the observed subtle changes in overall morphology cannot account for the 60% increase in inorganic content, further characterization was performed to ascertain how *C. sp.* responds to this increased mineral uptake. To this end the thickness of the valves was measured from TEM images specifically focusing on thickness variations of the raphe, porous area, and the overall valve in the presence of Al^3+^. TEM image analysis was carried out using an in-house MATLAB script which is presented in detail in the supplementary information Sect. 2 including Table S2 and Fig. S3. Figure 6a,b present the thickness maps of the whole valve and porous area of *C. sp.* grown at different Al^3+^ concentrations. This analysis clearly shows that the thickness of the valves increases with increasing Al^3+^ concentration (Fig. 6c). From C_0_ to C_3_ the thickness of raphe increased from 153 ± 12 to 188 ± 10 nm. The thickness of porous area (excluding the areolae) for C_0_, C_1_, C_2_, and C_3_ were 49 ± 7, 59 ± 6, 70.8 ± 5, 82 ± 8 nm, respectively. Increasing the Al^3+^ concentration to 2 µM resulted in an increase of valve thickness of approximately 40 nm (Fig. 6c). The increased thickness at nearly identical overall dimensions leading to an increase in the volume of silica making up the frustule as shown in Supplementary Table S3. According to the SEM images, the total volume of the *C. sp.* was approximately 1140 ± 10.2 µm^3^. The internal volumes and volumes of the frustule for the four cultures are depicted in Supplementary Table S3. There was approximately a 60% increase in the volume of silica making up the frustule by 2 µM Al^3+^ addition. These results confirmed that Al^3+^ addition led to Si uptake enhancement which ultimately increased the thickness and reduced the pore size.

### The effect of Al^3+^ on the internal structure of *C. sp*

**Figure 7:**
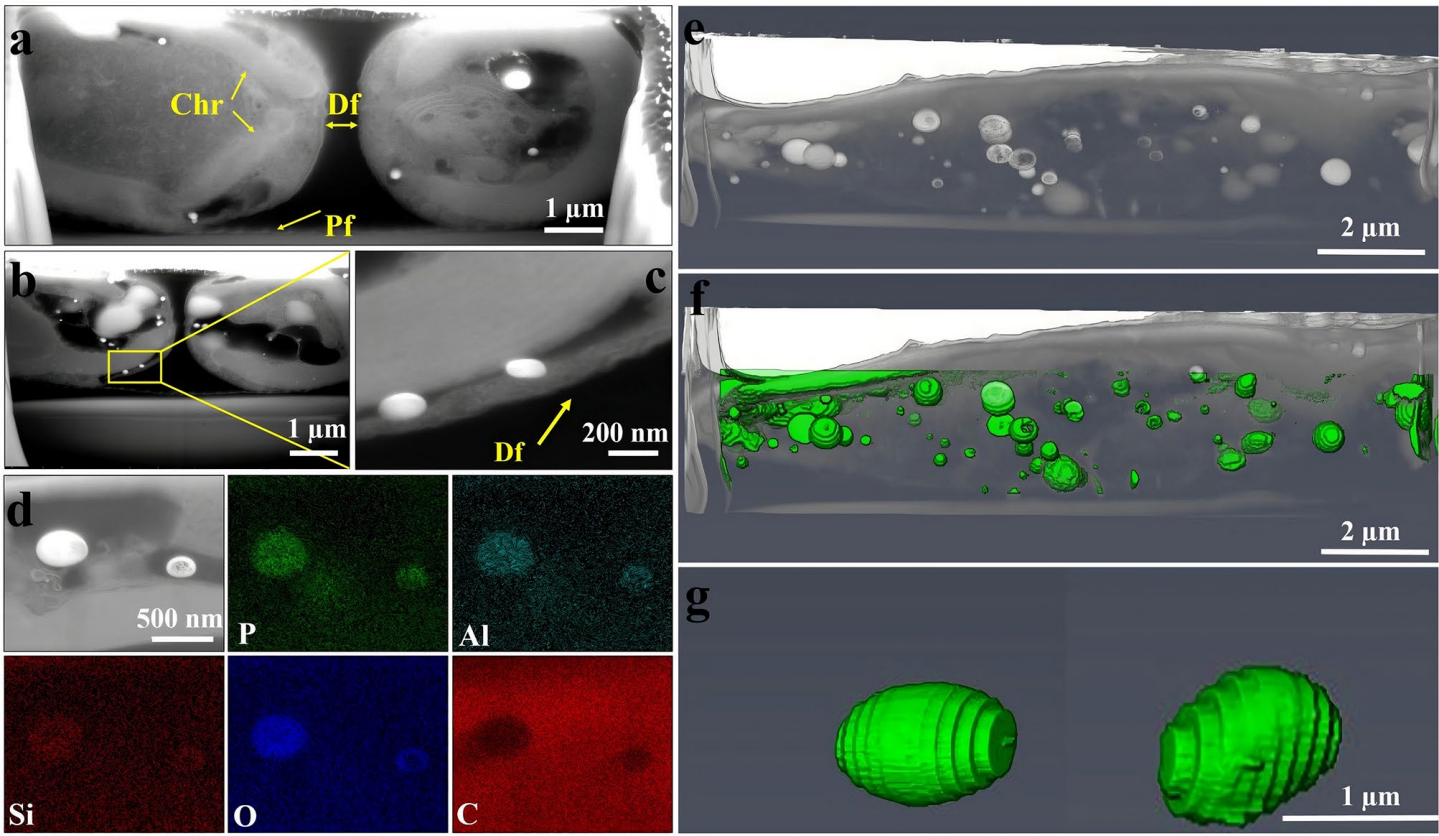
SEM images of FIB cross-sections of *C. sp.* cell grown in 2 µM Al^3+^. (**a**) Df = daughter frustules which are being formed within the cell, Pf = parental frustule*,* Chr = Chloroplasts*;* (**b**) presence of the particles at the different parts of the cell; (**c**) two particles are close to the region where the silica deposition vesicle (SDV) is located*.* (**d**) SEM image and EDS maps of the particles within the cell; (**e**–**g**) 3D rendering of the high contrast particles inside the cell.

In order to understand how *C. sp.* responds to Al^3+^, the internal structure of intact *P.* sp. was investigated using FIB-SEM tomography. By sequentially FIB sectioning and SEM imaging, 200 cross-sections of an entire diatom were imaged. SEM images of internal structures of C_3_ revealed the presence of high contrast particles in BSE mode within the cells (Fig. 7a–c). These particles were found in different regions of the cells. Figure 7a shows the internal structure of a parent cell in which two daughter cells are about to separate from each other. Chloroplasts (Chr) and newly formed valves (Df) are annotated in Fig. 7a. As shown in Fig. 7b,c most of the particles are found in the middle of the parent cell with some of them being close to the region where the silica deposition vesicle (SDV) is usually located^45^. EDS-mapping was performed on one of the cross-sections during the milling process to analyze the elemental composition. As shown in Fig. 7d, Phosphorous, Aluminium, Oxygen, and Silicon are the main elements found in the particles. A 3D rendering of the internal structure of a *C. sp.* cell highlighting the distribution of particles is shown in Fig. 7e–g. The high contrast particles are located at the different parts of the cell having a mean diameter and volume of 570 ± 300 nm and 0.22 ± 0.17 µm^3^, respectively. In contrast to C_3_, no high contrast particles were observed within the internal structure of C_0_ (Supplementary Fig. S9).

### The effect of Al^3+^ on silica hydrolysis

The most studied effect of Al^3+^ on diatom frustules is the reduction of the silica hydrolysis or dissolution rate^26^. Here, atomic absorption spectroscopy was employed to monitor the effect of Al^3+^ on the dissolution of *C. sp.* frustules in demineralized water. The released Si concentration of C_0_ and C_3_ as a function of time is provided in Supplementary Fig. S10. The results show that after 24 h the Si concentration of C_0_ reached 80 ± 6 µM. Interestingly, no increase in Si concentration was detected after this point. In contrast, for C_3_ only 3 ± 0.9 µM Si was detected after 24 h. The Figure shows that there has been a gradual increase in the Si concentration until day 6 where it reached 26 ± 4 µM. These findings provide further support for the hypothesis that Al incorporated frustules are remarkably hydrolysis resistant compared to their Al free types.

### Discussion

The results of this investigation demonstrate that Al^3+^ addition decreased *C. sp.* growth rates significantly while the width, length and area of the valves were not altered (Figs. 2 and 5a). This decrease in growth rate did not result in a concomitant decrease in Si uptake, indicating an increased intracellular Si concentration in the presence of Al^3+^. This increased silica uptake resulted in higher silica volume in the frustule (Table. S2) leading to an increased frustule thickness (Fig. 6). Hence, the inorganic content of *C. sp.* grown in the presence of 2 μM Al^3+^ as found by TGA appeared to be approximately 60% higher. The role of mineral particles inside the cell, most likely being metal–polyphosphate granules, has been suggested to be a tolerance mechanism for many organisms including diatoms to detoxify Al and other hard cations^28,46^. Therefore, the formation of the particles within the cell in the presence of Al^3+^ which contained a high amount of P, O, Si and Al (Fig. 7) could be a tolerance strategy for *C. sp.* to cope with Al^3+^. In accordance with the previous investigations, present findings have demonstrated that Al^3+^ can modify the frustule properties. For instance, the increase of the silica content of *Asterionella Ralfsii var.americana* with Al^3+^ addition is comparable to our results even though the reported valve size reduction was not observed in *C. sp.*^47^. Particularly the dissolution experiments, demonstrating a remarkable effect of Al^3+^ on the solubility of the frustule, are consistent with previous investigations^48^.

Notwithstanding the 60% increase in the silica/inorganic content in the presence of Al^3+^, *C. sp.* could completely regulate the frustule formation without changing the overall morphology. The reduced diameter of small pores, in contrast to the unmodified areolae, cross-extensions, and transapical ribs, imply that merely the delicate fine-structures of the valve were influenced by Al^3+^, as it has been observed for Ge and Ti incorporation into *C. sp.*^20,49^ as well. In addition, we have shown here that even 0.2 µM Al^3+^ (~ 500Si:1Al) remarkably impacted the thickness of the valves. It can thus be suggested that a higher Si uptake in the presence of Al^3+^ led to the accumulation of a higher amount of Si inside the SDV which resulted in a thicker valve with smaller pores. It is worth referring that the process of valve formation for raphid pennate diatoms such as *C. sp.* undergoes a sequence of distinct stages for various morphological features such as central nodule, raphe, cross extension, and transapical ribs^50^. Therefore, a possible explanation for the homogenous distribution of Al and Si in EDS mapping and enhancing the thickness of porous area and raphe is that Al^3+^ is involved throughout the different stages of the valve formation. Also, the results of Al^3+^ uptake, FIB-SEM, and ^27^Al solid state NMR support the assumption that Al^3+^ is internalized and associated with the silicification process in *C. sp.*, but further work is needed to show this unequivocally. 

**Figure 8:**
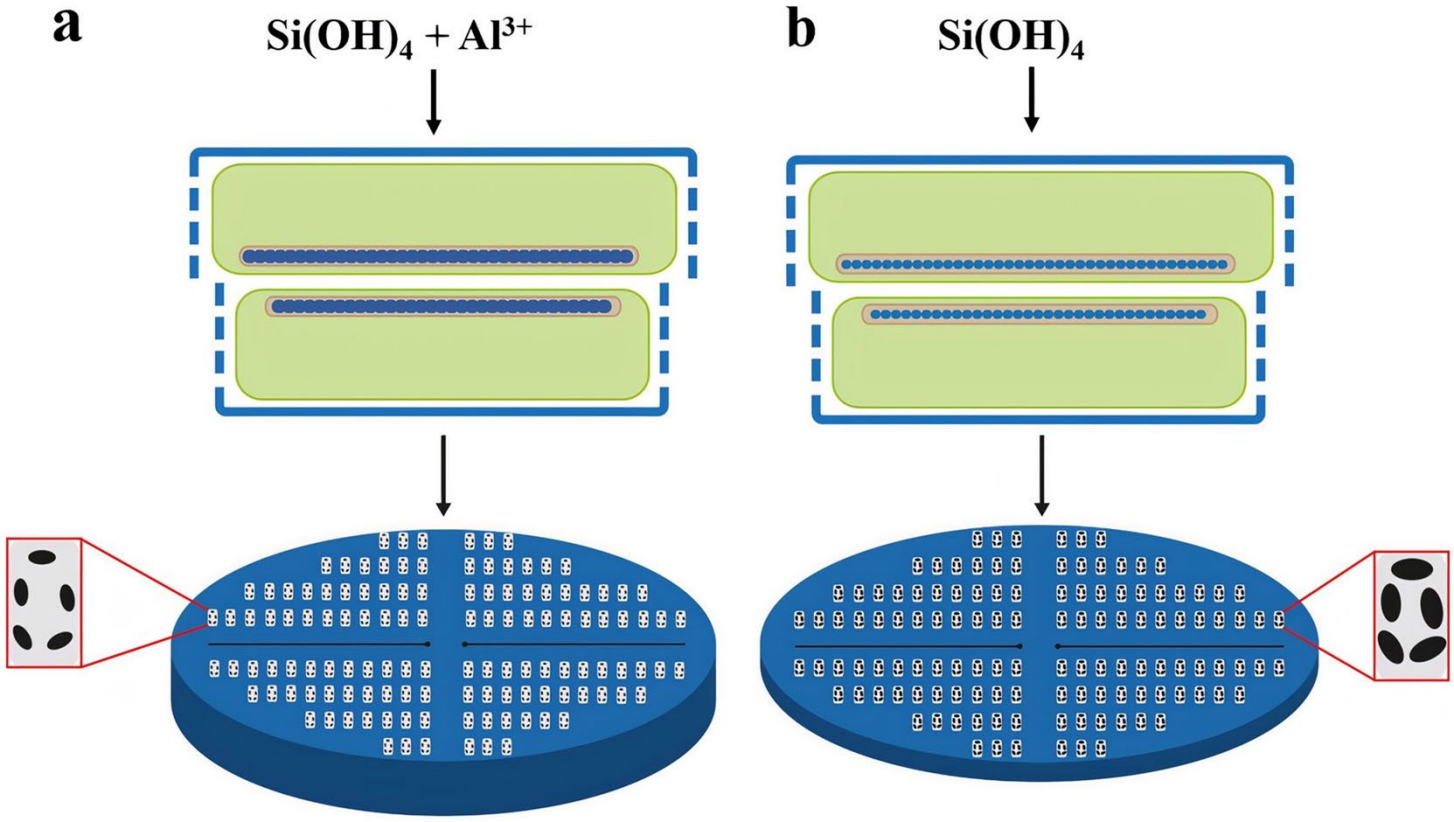
Feeding diatom cells with and without Al^3+^ solution. (**a**) Diatom cell takes up Al^3+^ and Si(OH)_4_ during the cell cycle division resulting in a higher silica content inside the SDV(dark blue color) and a new thick valve with smaller pores. (**b**) Absence of Al^3+^ in the growth medium leads to a thin valve with relatively large pores.

Previous studies have demonstrated that through chemical treatments such as acid etching or surface coating, the pore size and thickness of the frustule can be modified^3^. For instance, atomic layer deposition has been used to form an ultrathin film of TiO_2_ on the surface of the frustule of *Coscinodiscus sp.* and *Thalassiosira eccentrica* to modify the pore size and the thickness^51^. Besides, etching of the frustule of *Coscinodiscus* sp. with HF solution was performed to enlarge the pore size^52^. The main drawbacks of these methods are the involvement of extra steps after harvesting of the frustule and the use of high-risk chemicals. In contrast, modification of the frustule was effected with small amounts of metal salts during the cell growth of *C. sp.* in the present study. By changing the Al^3+^ concentration in the growth medium we could control the thickness and pore size of the frustule at the nanoscale (Fig. 8). The current study shows that employing naturally occurring concentrations of a nonessential element in the growth medium leads to the formation of diatom frustules with a tunable morphology (thickness and pore size) and composition.

In summary, diatom *C. sp.* responded to the presence of Al^3+^ by decreasing the growth rate, formation of particles inside the cell, and increasing the intracellular silica content which resulted in thicker valves with smaller pores. The pore size, thickness, and dissolution rate of the valves of *C. sp.* can be tuned by Al^3+^ concentration in the growth medium. In addition, uptake and incorporation of Al^3+^ into the frustule resulted in its homogenous distribution at the submicron level. While the underlying mechanisms is not clear, similar approaches and different species might be used to synthesize materials with complex and well defined morphological, and potentially chemical and mechanical properties in the future.

## Supplementary information

Supplementary Information 1.

## Data Availability

The datasets generated during and/or analysed during the current study are available from the corresponding authors upon reasonable request.
