# Author Correction: Modifying the thickness, pore size, and composition of diatom frustule in *Craspedostauros sp.* with Al^3+^ ions

**DOI:** 10.1038/s41598-022-08154-8

**Published:** 2022-03-09

**Authors:** Mohammad Soleimani, Luco Rutten, Sai Prakash Maddala, Hanglong Wu, E. Deniz Eren, Brahim Mezari, Ingeborg Schreur‑Piet, Heiner Friedrich, Rolf A. T. M. van Benthem

**Affiliations:** 1 Laboratory of Physical Chemistry, and Center for Multiscale Electron Microscopy, Department of Chemical Engineering and Chemistry, Eindhoven University of Technology, Groene Loper 5, 5612 AE Eindhoven, The Netherlands; 2 Laboratory for Inorganic Materials and Catalysis, Department of Chemical Engineering and Chemistry, Eindhoven University of Technology, P.O. Box 513, 5600 MB Eindhoven, The Netherlands; 3 Institute for Complex Molecular Systems, Eindhoven University of Technology, Groene Loper 5, 5612 AE Eindhoven, The Netherlands

Correction to: *Scientific Reports* https://doi.org/10.1038/s41598-020-76318-5, published online 11 November 2020

The original version of this Article contained a repeated error, where the algae species *“Craspedostauros sp.”* was incorrectly referred to as “*Pinnularia sp*.” Furthermore, all references to *“C.sp.”* were incorrectly given as *“P.sp.”*

These errors have now been corrected in the original version of the Article and ts accompanying Supplementary Information file. The original Supplementary Information 1 file is provided below.

## Supplementary Information

Supplementary Information.

