# Supplementary material for: Author Correction: Modifying the thickness, pore size, and composition of diatom frustule in Craspedostauros sp. with Al3+ ions
Source: Sci Rep. 2022 Mar 9;12:4175. doi: 10.1038/s41598-022-08154-8 (PMC8907179; doi:10.1038/s41598-022-08154-8)
Supplement: Supplementary file 1 — Supplementary Information. [file 41598_2022_8154_MOESM1_ESM.pdf]

Supplementary Information

Modifying the thickness, pore size, and composition of diatom frustule in *Pinnularia sp.* with Al^{3+} ions

Mohammad Soleimani[1], Luco Rutten[1], Sai Prakash Maddala[1], Hanglong Wu[1], E. Deniz Eren[1], Brahim Mezari[2], Ingeborg Schreur-Piet [1], Heiner Friedrich[1,3*] & Rolf A. T. M. van Benthem[1*]

[1] Laboratory of Physical Chemistry, and Center for Multiscale Electron Microscopy, Department of Chemical Engineering and Chemistry, Eindhoven University of Technology, Groene Loper 5, 5612 AE, Eindhoven, the Netherlands
[2] Laboratory for Inorganic Materials and Catalysis, Department of Chemical Engineering and Chemistry, Eindhoven University of Technology, P.O. Box 513, 5600 MB Eindhoven, the Netherlands
[3] Institute for Complex Molecular Systems, Eindhoven University of Technology, Groene Loper 5, 5612 AE, Eindhoven, the Netherlands

*Corresponding authors:
Heiner Friedrich. Email: H.Friedrich@tue.nl
Rolf A. T. M. van Benthem. Email: r.a.t.m.v.benthem@tue.nl

1. Mean diameter of areolae and small pores measurements

The mean diameter of areola was measured for 15 valves per culture in SEM micrographs using an in house MATLAB script[1]. Supplementary Figure S1 exhibits the selected axes for each areola. The average of the two-axes was used to determine areola diameter and error bars represent the standard deviations. Also, the mean diameter of small pore was determined via an in-house MATLAB script. Supplementary Figure S2 shows the procedure for determining the mean dimeter of small pore via the Matlab program.

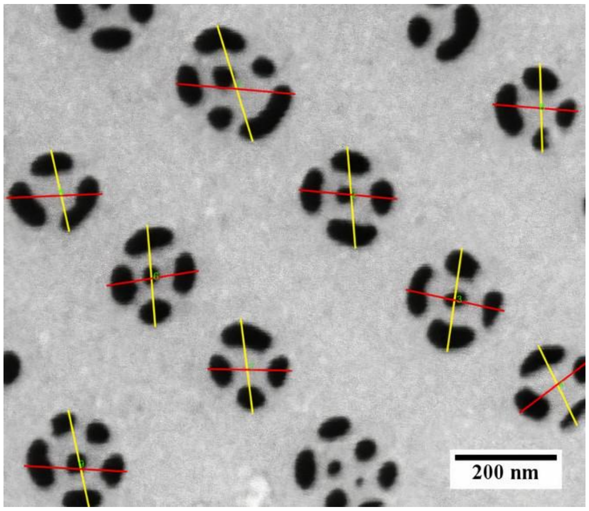

Supplementary Figure S1. Selected axes for measuring the diameter of the areolae.

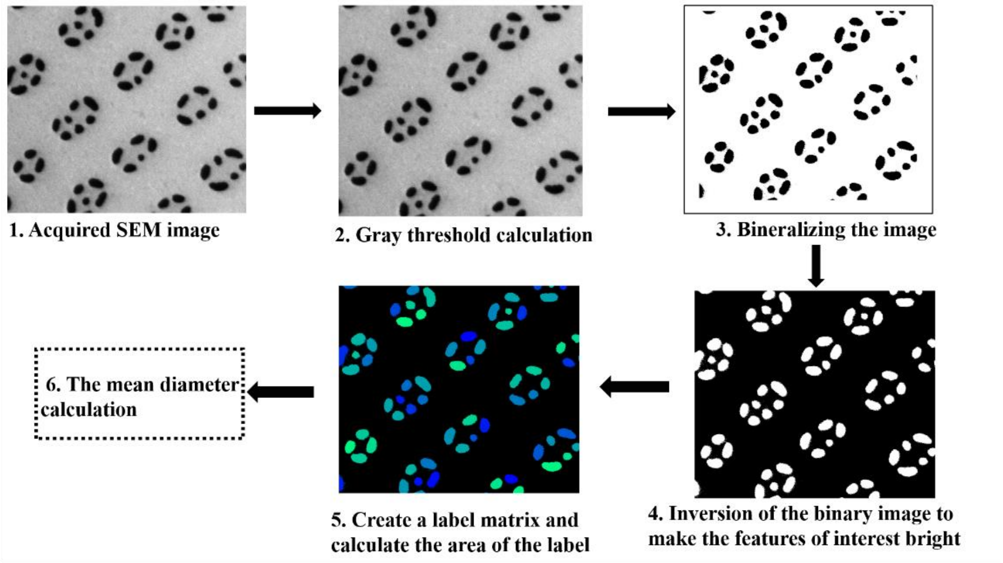

Supplementary Figure S2. Image analysis process for measuring the mean diameters of small pores.

2. Thickness mapping

The thickness of the valves was measured from TEM images in the following manner: The contrast in the TEM images can be approximated by mass-thickness contrast considerations. Diatoms frustules, are amorphous materials, loaded on the continuous carbon grid and were imaged at a low magnification (800×). To map the thickness of a single diatom, one flat field (FFD) TEM image only containing the unscattered incident electron flux I_0 and one TEM image containing the electron flux I_t transmitted through the sample were acquired. The thickness of diatoms t_D can be estimated (Equation (1)) based on Lambert-Beer Law:

$$\frac{I_t}{I_0} = \exp\left(-\left(\frac{t_\mathrm{C}}{\Lambda_\mathrm{C}} + \frac{t_\mathrm{D}}{\Lambda_\mathrm{D}}\right)\right) \quad (1)$$

Where t_C is the thickness of the continuous carbon film in the TEM grid, and Λ_C and Λ_D are the elastic mean free path (EMFP, Λ) of the carbon and the diatom, respectively. EMFP calculations are based on Reimer's book[2]. All the thickness analysis and EMFP calculations were performed using in-house MATLAB scripts[3,4]. Important parameters used in the calculations were shown in Supplementary Table S2. Mapping the local thickness of the diatom was carried out using in-house MATLAB scripts. The detailed image analysis procedures are shown in Supplementary Figure S3.

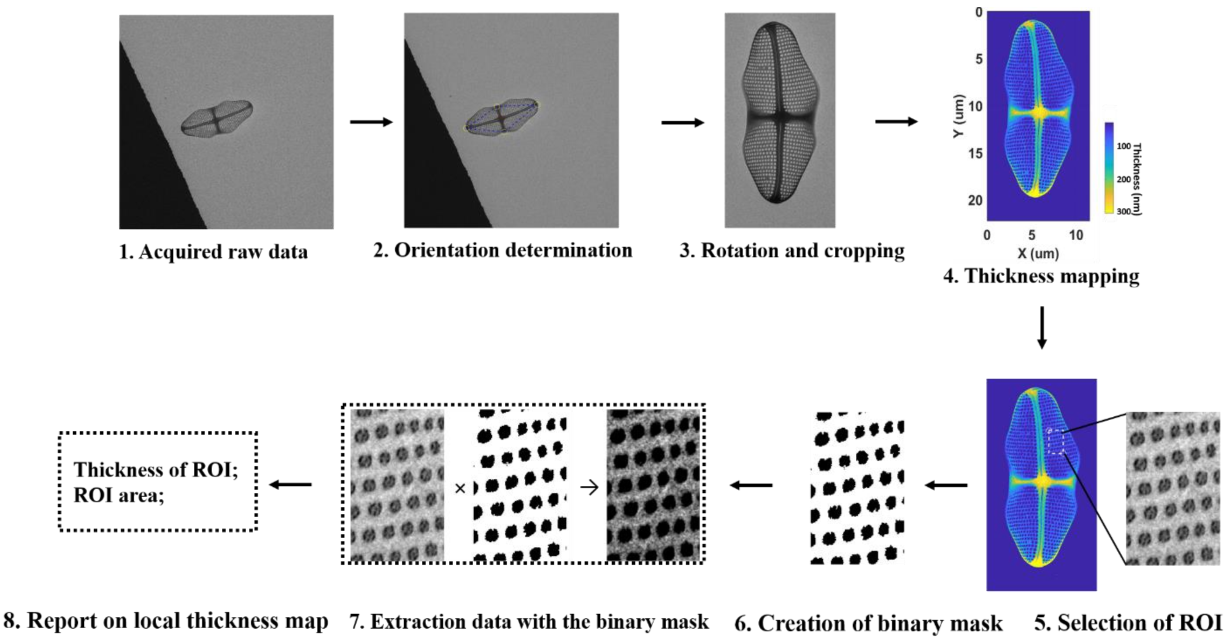

Supplementary Figure S3. Image-processing procedures for single diatom and local area thickness mapping.

3. Internal volume of *P.sp* and the frustule volume

The volume of the entire *P.sp* was calculated using a simple assumption that *P.sp* is a rectangular prism. V= L×W×H. Where V, L, W, and H are volume, length, width and height of the whole *P.sp* (Supplementary Figure S4). Since, the length and width of the valves of the four cultures were comparable (SEM images) we used an average of *P.sp* dimensions for determining the entire volume. The internal volume was calculated by subtracting the thickness of the frustule from the dimensions of the whole *P.sp*. Therefore, the volume of silica making up the frustule for each culture is the difference between the total volume and internal volume (Supplementary Table S3). It is worth noting that for this calculation, the thickness of the porous area was used as the thickness of the whole frustule.

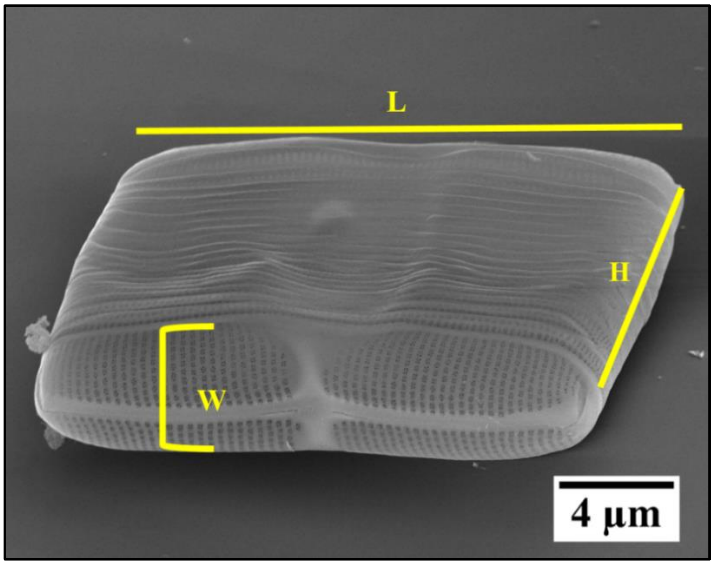

Supplementary Figure S4. Overall structure of an intact *P.sp* cell. Length, width, and height are shown.

4. FIB-SEM process of the embedded *P.sp* cell

The embedded *P.sp* cell was positioned at Eucentric height (10 mm) and tilted to 52° with the intention that the electron beam and the ion beam are focused at the coincidence point. Before the Serial Slice and View (SSV), a protective layer of platinum with dimensions of 20×10 ×1 µm was deposited on the surface of *P.sp* using ion beam deposition at 30 keV and an ion beam current (IBC) of 0.3 nA. In order to remove material in front of the embedded cell, bulk milling was performed with dimensions 40× 20 × 10µm (IBC = 15 nA). Side trenches of 10× 20 × 5µm were created using an IBC of 7 nA. The fiducial marker was made with z ≥1µm (IBC = 3nA). Next the electron beam was focused on the cleaned block face and automated SSV operation was initiated using a milling IBC of 1 nA and a slice thickness of either 50 nm or 100 nm. Images of new revealed surface were taken in BSE mode.

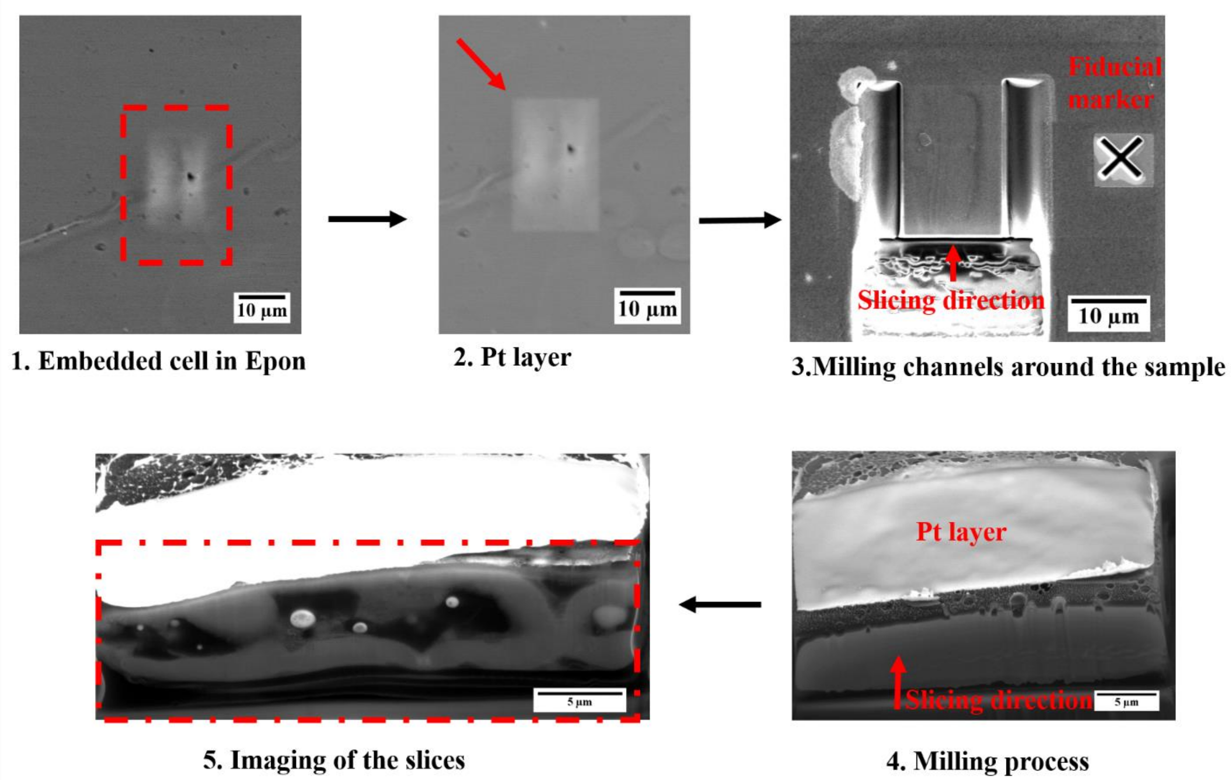

Supplementary Figure S5. FIB-SEM process steps.

5. SEM-EDS measurements of C_0-C_3

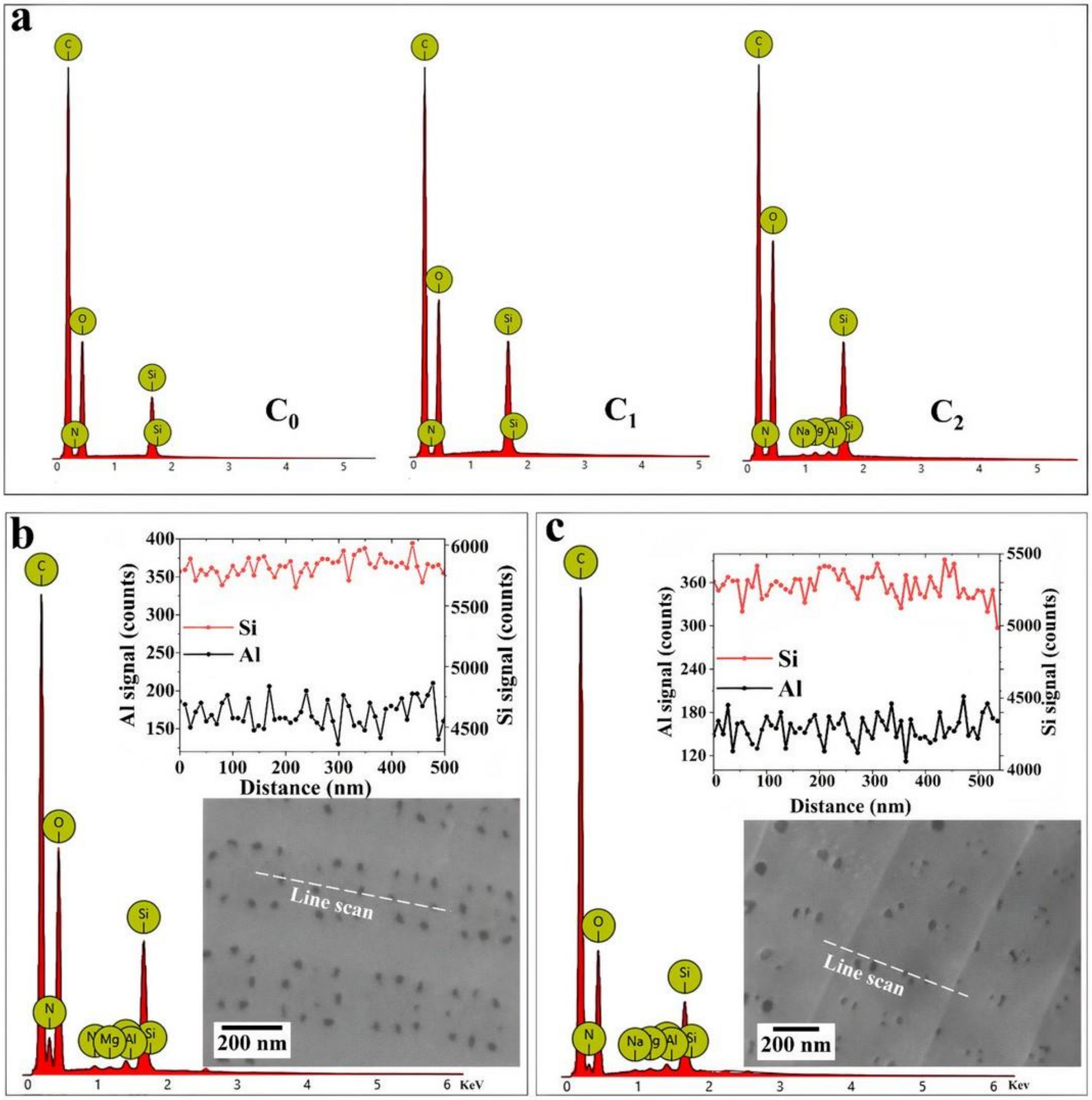

Supplementary Figure S6. (a) SEM-EDS spot measurements of C_0-C_2; (b) Spot and line scan of porous are of C_3; (c) Spot and line scan of girdle bands of C_3.

6. Si and Al^{3+} uptake comparison

As shown in Supplementary Figure S7, Si and Al^{3+} were taken up during the cultivation time. the concentration of both elements decreased as a function of time. During the first 48 hours, the relative amounts of consumed Si and Al^{3+} were comparable. However, after this point, a the rate of uptake differed.

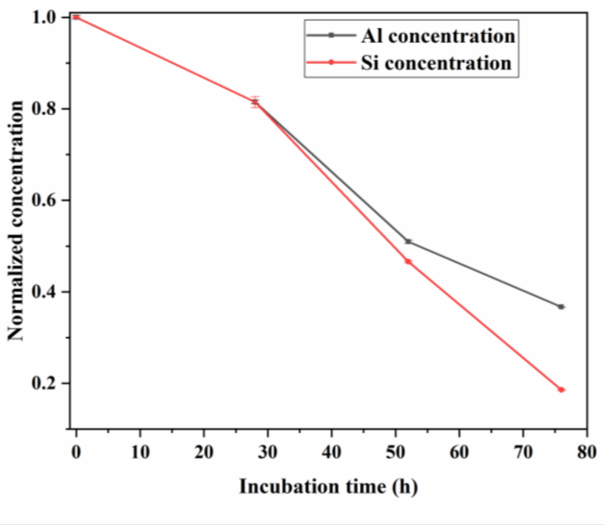

Supplementary Figure S7. Si and Al^{3+} uptake comparison of C_3 as a function of time

7. Control experiment: Variation of Si concentration in diatom free culture

Artificial seawater supplemented with f/2 medium (ASW-f/2) and 2 μM Al^{3+} was incubated in a culture flask in the absence of *P.sp* cells. Every day, a sample was taken, and Si concentration was determined via atomic absorption spectrometer. As shown in the Supplementary Figure S8 there was no reduction of Si concentration during the cultivation period.

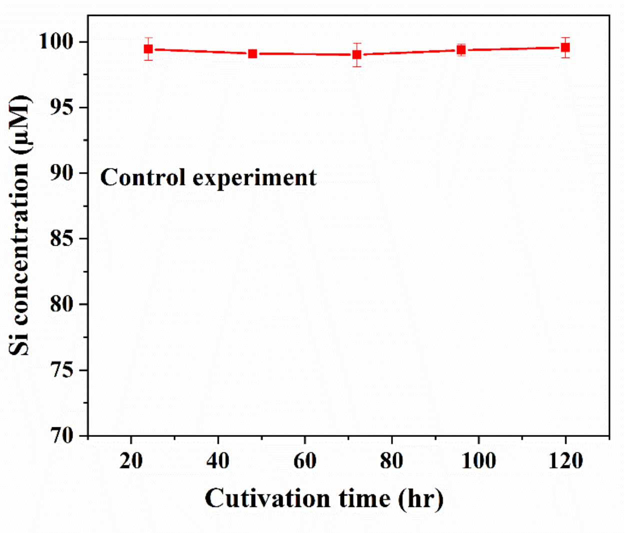

Supplementary Figure S8. Control experiment without *P.sp* cells.

8. Investigation of the internal structures of C_0 using FIB-SEM

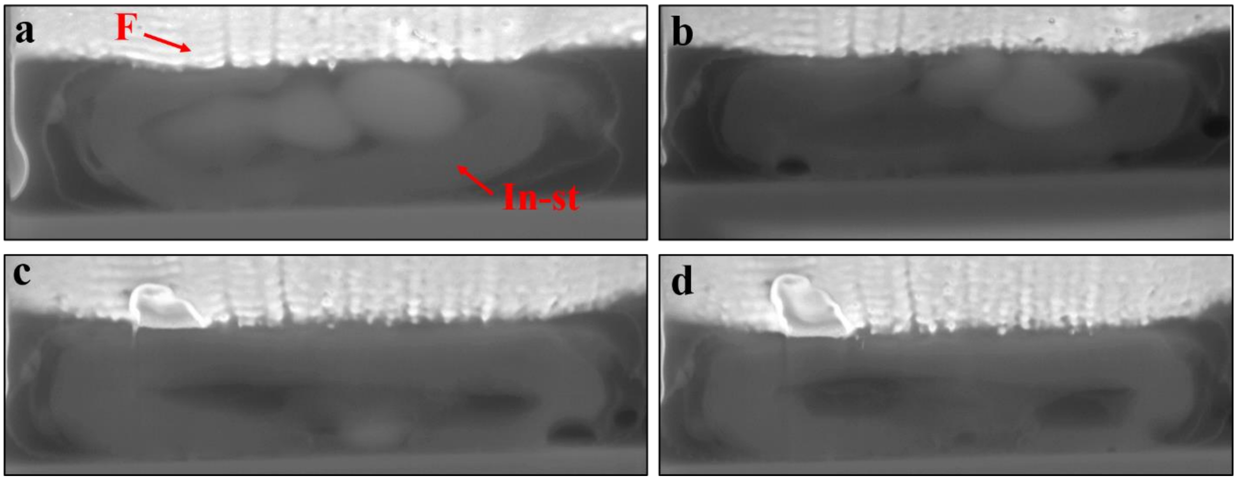

Supplementary Figure S9. (a-d) depict the internal structures of C_0 during the slice and view process. F= frustule, In-st= internal structures.

9. The effect of Al^{3+} on silica hydrolysis

Diatoms (C_0 & C_3) were stored in demineralized water at 90 °C for 6 day. The Si concentration was determined via atomic absorption spectrometry.

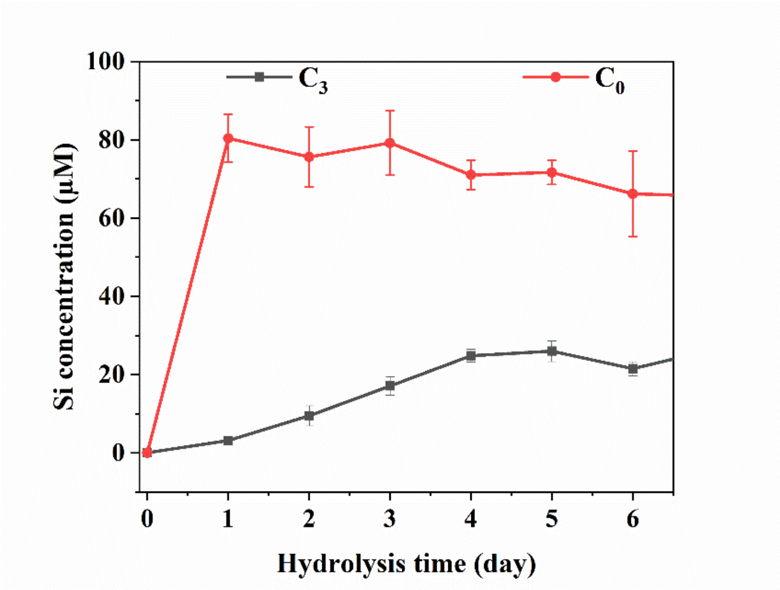

Supplementary Figure S10. Si concentration as a function of time for C_0 and C_3. Error bars represent standard deviation.

Supplementary Table S1. Chemical composition of artificial seawater supplemented with f/2 medium.

**Chemical**	**Final concentration**
NaCl	356 mM
Na_2SO_4	25 mM
$CaCl_2.2H_2O$	8 mM
KCl	8 mM
H_3BO_3	404 µM
KBr	714 µM
NaF	71 µM
$NaHCO_3$	1.2 mM
$MgCl_2.7H_2O$	47 mM
$Na_2SiO_3.9H_2O$	106 µM
$NaH_2PO_4.H_2O$	36 µM
$NaNO_3$	880 µM
$Fe(NH_4)_2(SO_4)_2.\ 6H_2O$	11.7 µM
$Na_2MoO_4.2H_2O$	35.9 nM
$ZnSO_4.7H_2O$	80 nM
$CuCl_2$	40 nM
$CoSO_4.\ 7H_2O$	50 nM
$MnSO_4.4H_2O$	0.9 µM
$Na_2EDTA.2H_2O$	11.7 µM
Biotin vitamin	1 ml of a 2 mg/ml solution
vitamin B_{12}	1 ml of a 4 mg/ml solution
Thiamine vitamin	1 ml of a 100 mg/ml solution

Supplementary Table S2. Parameters used in EMFP (Λ) calculations.

Materials	M_w, g/mol	ρ, g/cm^3	Λ, nm
$SiO_{2(amorphous)}$	60.08	2.00	214.78
Carbon film	12.00	2.10	282.54

Supplementary Table S3: Internal volume of *P.sp* cell and the volume of silica making up the frustule (frustule volume). Average $\pm$ Standard Deviations (n=5).

**Culture number**	**Internal volume (μm^3)**	**Frustule volume (μm^3)**
C_0	1121.7±2.3	19.2±3.7
C_1	1119.6±3.4	21.4 ±1.5
C_2	1114.7±1.8	26.8±3.1
C_3	1110.2±2.7	30.2 ±3.4

Supplementary Table S4. Statistical data of SEM and TEM image analyses with n = number of measured valves per culture and expressed as Average $\pm$ Standard Deviations.

**Sample**	**C_0**	**C_1**	**C_2**	**C_3**
**Features size**				
Valve length μm (n=15)	19.2±2.9	19.6±2	19.5±2.6	19.4±3.1
Valves width μm (n=15)	6.9±0.8	7.1±0.6	7.1±0.9	6.97±1
Valve area μm^2 (n=15)	99.1±12	101.5±13	100.4± 16	100.3±15
Mean diameter of areola nm (n=15)	189±9	182±8	189±27	182±20
Mean diameter of small pore nm (n=15)	70±9	61±10	40±5	35±4
Width of transapical rib nm (n=15)	190 ±38	191±24	193±19	188±23
Width of cross extension nm (n=15)	105±19	110±16	108±26	110±19
Distance between neighboring areolae within a pore array nm (n=15)	278±14	272±15	273±12	277±15
Distance between two areolae of two parallel arrays nm (n=15)	346 ±24	349±19	349±30	347±18
Mean thickness of porous area nm (n=5)	49±7	59±6	71 ±5	82±8
Mean thickness of raphe nm (n=5)	153± 17	175±15	178±20	188±18
Mean thickness of valve nm (n=5)	83±7	101±3	108±2	121±10

References

1 Mirabello, G. *et al.* Understanding the formation mechanism of magnetic mesocrystals with (cryo-) electron microscopy. *Chemistry of Materials* **31**, 7320-7328 (2019).

2 Kohl, H. & Reimer, L. *Transmission electron microscopy: physics of image formation*. (Springer, 2008).

3 Su, H. *et al.* Growth kinetics of cobalt carbonate nanoparticles revealed by liquid-phase Scanning transmission electron microscopy. *The Journal of Physical Chemistry C* **123**, 25448-25455 (2019).

4 Ianiro, A. *et al.* Liquid–liquid phase separation during amphiphilic self-assembly. *Nature Chemistry* **11**, 320-328 (2019).
